# Effects of 4 Weeks of High-Definition Transcranial Direct Stimulation and Foot Core Exercise on Foot Sensorimotor Function and Postural Control

**DOI:** 10.3389/fbioe.2022.894131

**Published:** 2022-06-01

**Authors:** Songlin Xiao, Baofeng Wang, Xini Zhang, Junhong Zhou, Weijie Fu

**Affiliations:** ^1^ Key Laboratory of Exercise and Health Sciences of Ministry of Education, Shanghai University of Sport, Shanghai, China; ^2^ The Hinda and Arthur Marcus Institute for Aging Research, Hebrew SeniorLife, Boston, MA, United States; ^3^ Harvard Medical School, Boston, MA, United States; ^4^ School of Kinesiology, Shanghai University of Sport, Shanghai, China; ^5^ Shanghai Frontiers Science Research Base of Exercise and Metabolic Health, Shanghai University of Sport, Shanghai, China

**Keywords:** high-definition transcranial direct current stimulation, foot core exercise, foot sensorimotor function, toe flexor strength, passive ankle kinesthesia, postural control

## Abstract

**Objective:** This study aimed to examine the effects of 4 weeks of high-definition transcranial direct current stimulation (HD-tDCS) and foot core exercise (FCE) on foot sensorimotor function (i.e., toe flexor strength and passive ankle kinesthesia) and postural control.

**Methods:** In total, 36 participants were randomly assigned into three groups as follows: HD-tDCS, FCE, and the control group. A total of 12 training sessions were performed over 4 weeks (i.e., three sessions per week) in the laboratory. The HD-tDCS group received 20-min HD-tDCS with a current density of 2 mA, and the FCE group completed short foot exercise, towel curls, toe spread and squeeze, and balance board training. Participants in the control group just maintained the activities what they usually did and did not receive any interventions. Foot muscle strength, passive ankle kinesthesia, and postural control were assessed at baseline and post-intervention.

**Results:** HD-tDCS induced a greater decrease in the percentage changes in the passive kinesthesia thresholds of ankle inversion (*p* < 0.001) and eversion (*p* = 0.013) than the control group. Compared with the control group, a significant increase in the percentage change in the metatarsophalangeal joint flexor strength was found in the HD-tDCS group (*p* = 0.008) and the FCE group (*p* = 0.027), and a significant increase in the percentage change in toe flexor strength was observed in the FCE group (*p* = 0.015). Moreover, FCE induced a greater reduction in the percent changes in the medial–lateral average center of gravity sway velocity in one-leg standing with eyes open (*p* = 0.033) and the anteroposterior average center of gravity sway velocity in one-leg standing with eyes closed (*p* < 0.001) than control.

**Conclusion:** This study demonstrated that 4 weeks of HD-tDCS and FCE induced distinct benefits on foot sensorimotor function and the standing postural control performance in healthy young adults. HD-tDCS could improve the metatarsophalangeal joint flexor strength and the passive kinesthesia thresholds of ankle inversion and eversion. Meanwhile, FCE could also enhance foot muscle strength and enhance postural control performance in one-leg standing.

## Introduction

The foot core system consists of a complex foot structure, including active, passive, and neural subsystems, providing stability and flexibility when coping with changing foot demands (McKeon et al., 2015). During daily weight-bearing activities (e.g., walking), the foot functions by performing force attenuation with loading, transmitting force during propulsion, providing afferent information to accommodate uneven terrain, and maintaining postural control (Kelly et al., 2014; Fraser and Hertel, 2019). The diminished sensorimotor function of the foot and ankle has been linked to poor functional performance and associated with loading-related injuries (Ridge et al., 2019; Kakouris et al., 2021). For example, weakness of the intrinsic foot muscles suggested that inefficient active support of the arch may contribute to injuries, such as plantar fasciitis (Wearing et al., 2006; van Poppel et al., 2021). Thus, strengthening the foot sensorimotor function is critical for addressing degenerative foot-related conditions and maintaining the intact capacity for functional independence in daily activities.

One commonly used strategy of strengthening the foot sensorimotor function is foot core exercise (FCE), which is traditionally described as intrinsic foot muscle flexion exercises, such as towel curls, toe spread-out exercise, and short foot exercise (Lynn et al., 2012; Sulowska et al., 2016; Fraser and Hertel, 2019). Previous studies demonstrated that FCE was able to improve ankle-foot functional performance by activating the plantar intrinsic muscles (Mulligan and Cook, 2013; Hashimoto and Sakuraba, 2014). For example, Mulligan et al. reported that 4 weeks of short foot exercise decreased navicular drop, increased the arch foot index, and improved motor performance during a static unilateral balancing activity in young healthy adults (Mulligan and Cook, 2013).

Moreover, the regulation of the foot core system also depends upon the activation of supraspinal networks, such as the brain cortical networks. Studies have shown the execution of foot movements was associated with the range and magnitude of activation in the sensorimotor cortex including the primary motor cortex (M1), primary sensory cortex (S1), and supplementary motor area (Orr et al., 2008). Nowadays, noninvasive brain stimulation techniques are used to enhance physical performance in healthy individuals, and transcranial direct current stimulation (tDCS) represented the most widely used technique (Angius et al., 2018). Generally, tDCS induced a weak electric direct current applied to the scalp to modulate cortical excitability to improve physical performance (Nitsche and Paulus, 2001). In particular, an early study showed that one session of 20-min tDCS applied over the sensorimotor cortex could enhance toe pinch force in young healthy adults (Tanaka et al., 2009). A recent systematic review has shown that tDCS could ultimately influence the neural circuitry responsible for the neuromechanical regulation of the foot and ankle and then improve their muscle strength and somatosensory function, which indicated that targeting the excitability of the cortical regions may help further augment the foot sensorimotor function (Xiao et al., 2020b). More importantly, such positive effects induced by tDCS may be accumulated in multiple-session interventions to improve performance by remarkably reshaping neuroplasticity (Nitsche et al., 2005). However, the effects of multi-session tDCS on the foot sensorimotor function have not been well examined, especially compared with FCE.

Therefore, this study aimed to examine the effects of 4 weeks of FCE and multi-session high-definition tDCS (HD-tDCS) on foot sensorimotor function and postural control. This study also hypothesized that 4 weeks of FCE and HD-tDCS intervention could result in increasing the foot muscle strength, passive ankle kinesthesia, and postural control as compared with control.

## Materials and Methods

### Participants

The sample size was calculated using *a priori power analysis* with a statistical power of 0.80, a probability level of 0.05, and an effect size f of 0.38 (Yotnuengnit et al., 2018) *via* G*Power 3.1.9.2 software. In total, 10 participants per group were identified as achieving sufficient power. Thus, to account for up to 20 percent attrition, 36 young healthy male adults without the habit of regular exercise were recruited from a university community *via* advertisements and randomly assigned to three groups (*n* = 12 in each group, Table 1) by using a Microsoft Excel random number table. The inclusion criteria were as follows: no history of lower extremity injuries in the past 6 months and not participating in other training programs of tDCS or FCE. The exclusion criteria were as follows: skin allergies and any contraindications to the use of tDCS (e.g., metal-implanted devices in the brain). All participants provided written informed consent as approved by the Institutional Review Board of the Shanghai University of Sport (No.102772021RT035).

**TABLE 1:** Participant demographics.

Group	Age (years)	Height (cm)	Weight (kg)
HD-tDCS group (*n* = 12)	21.9 ± 2.1	174.9 ± 6.1	69.8 ± 7.6
FCE group (*n* = 12)	22.7 ± 2.0	173.5 ± 7.1	69.3 ± 13.1
Control group (*n* = 12)	23.5 ± 1.5	177.5 ± 6.1	75.2 ± 7.1
*P*	0.135	0.272	0.256

### Intervention

Given the duration of the intervention in the previous studies (Lynn et al., 2012; Yotnuengnit et al., 2018), participants received a total of 12 training sessions over 4 weeks (i.e., three sessions per week) in the laboratory in both HD-tDCS and FCE groups. Within each week, at least 1 day of resting was provided between sessions.

**FIGURE 1:**
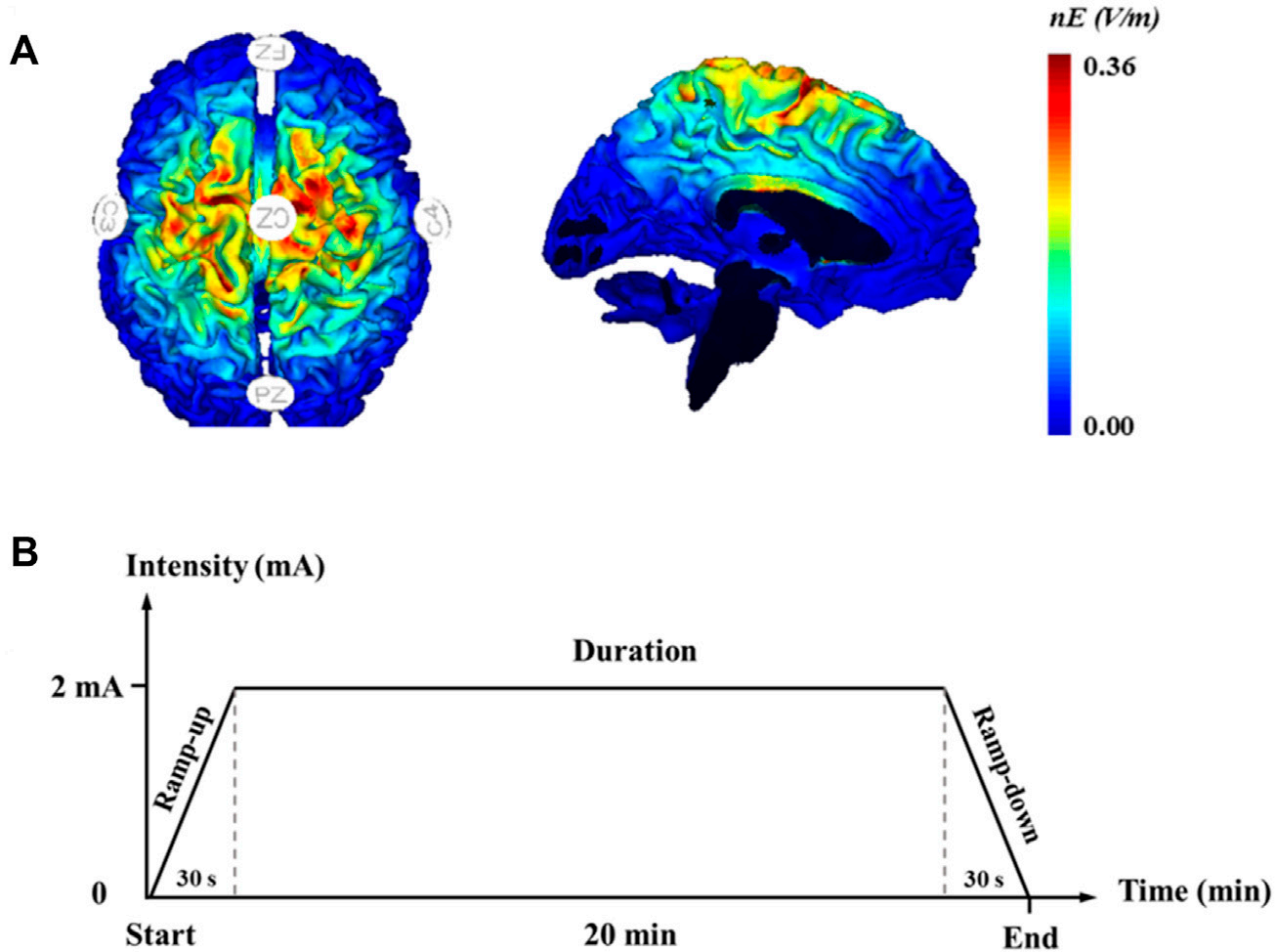
High-definition transcranial direct current stimulation. **(A)** Electrode montages for tDCS and simulated distribution of electrical field in the brain; **(B)** high-definition transcranial direct current stimulation protocol.

The participants in the HD-tDCS group received HD-tDCS, and they were asked not to imagine any foot movements. A 4 × 1 ring-type high-definition tDCS was administered with a battery-driven, wireless multichannel Starstim^®^ neurostimulator system (Neuroelectrics, Barcelona, Spain). The current was delivered using round gel Ag/AgCl electrodes (3.14 cm^2^). The anodal electrode was placed over the Cz and surrounded by four return electrodes (i.e., C3, C4, Fz, and Pz) on the basis of 10/20 electroencephalogram brain templates (Figure 1A) (Xiao et al., 2020a). The maximum current intensity of this HD-tDCS was set at 2 mA, and the stimulation duration was 20 min. Neuro-modeling results showed that a high and focal electric field induced by this montage effectively penetrated the sensorimotor cortex located along the longitudinal fissure controlling the foot-ankle area (Ma et al., 2020). The current intensity was ramped up from 0 to 2 mA in the initial 30 s at the beginning of the stimulation and ramped down to 0 mA in the last 30 s of the stimulation, as shown in Figure 1B (Boonstra et al., 2016). The participants were asked to complete a questionnaire at the end of each stimulation visit to evaluate the potential side effects.

**FIGURE 2:**
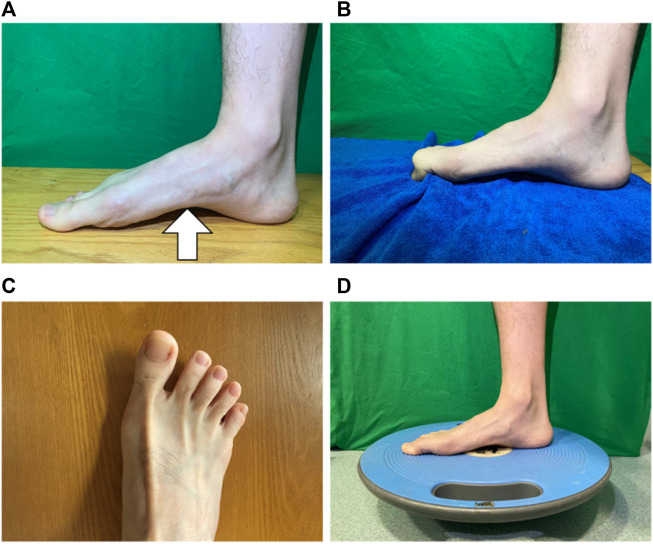
Foot core exercise. **(A)** short foot exercise; **(B)** towel curls; **(C)** toe spread and squeeze; and **(D)** balance board training.

For the FCE group, the training sessions consisted of short foot exercise, towel curls, toe spread and squeeze, and balance board training (Figure 2), with the goal to strengthen the intrinsic and extrinsic foot muscles and the functionalities of the foot and ankle (Table 2) (Ridge et al., 2019). All participants were verbally instructed, provided with a demonstration, and guided through a single practice trial. Following the instruction, the participants sequentially performed each exercise to the best of their ability barefoot, and one session was completed within 20 min. The participants in the control group did not receive any intervention.

**TABLE 2:** Foot core exercise progression.

Training	Week 1	Week 2	Week 3	Week 4
Short foot exercise	two sets of 10 times	two sets of 15 times	two sets of 20 times	three sets of 20 times
Towel curls	three sets of 10 times	three sets of 20 times	three sets of 10 times (0.25 kg)	three sets of 15 times (0.5 kg)
Toe spread and squeeze	two sets of 10 times	two sets of 15 times	two sets of 20 times	three sets of 20 times
Balance board training	two sets of 20 s	two sets of 25 s	two sets of 30 s	three sets of 30 s

### Data Collection

Foot muscle strength, passive ankle kinesthesia, and postural control were assessed at baseline and post-intervention.

The passive kinesthesia threshold of the ankle joint was assessed using an ankle proprioception tester (KP-11, Toshimi, Shandong, China) (Sun et al., 2015). In particular, each participant sat on an adjustable seat with their eyes closed, and the dominant foot was placed on the bottom of the foot pedal. The platform was randomly activated to drive the participant’s ankle in plantarflexion, dorsiflexion, inversion, and eversion. After the trigger and the direction of foot movement were confirmed, the participants pressed the stop button immediately. The ankle proprioception testing system showed the angle of the ankle, and then, the research personnel recorded it (i.e., the passive kinesthesia threshold of the ankle joint). The participants completed three trials of the test in each movement direction (i.e., plantarflexion, dorsiflexion, inversion, and eversion) in a randomized order.

The metatarsophalangeal joint (MPJ) flexor strength was measured using an MPJ flexor strength testing system customized and validated by the team (Zhang et al., 2019; Xiao et al., 2020b). Each participant was seated in the system with bare feet. The position and height of the seat were adjusted to make the thighs parallel to the ground, and the knee joint was fixed at 90°. The participants were asked to flex the MPJ and press the pedal for 10 s with maximum force. The peak MPJ flexor strength (N) was then obtained and normalized by the bodyweight of each participant.

The toe flexor strength was measured in a sitting position by using a toe grip dynamometer (T.K.K.3361, Niigata, Japan) (Kurihara et al., 2014; Yamauchi and Koyama, 2015). Each participant was asked to sit on an adjustable seat, and the dominant foot was placed on the dynamometer and fixed with the heel stopper. The participants were asked to flex their toes vigorously for at least 3 s. The peak toe flexor strength (N) was recorded and normalized by the bodyweight of each participant.

In the postural control test, each participant stood barefoot on the balance testing system (Super Balance, Acmeway, Beijing, China) (Xiao et al., 2020a). All participants completed three trials within each of the following conditions: one-leg standing with eyes open (OL_EO) and eyes closed (OL_EC) for 20 s. The system recorded the sway velocity of the CoG in the medial–lateral (ML) and anteroposterior (AP) directions.

### Statistics

SPSS 22.0 (SPSS Inc., Chicago, IL, United States ) was used to complete the statistical analysis of the training effects for percentage change from baseline to post-intervention during the groups, and all data were expressed by mean ± standard deviation. The Shapiro–Wilk test was used to examine if the outcomes were normally distributed, and Levene’s test was used to check homoscedasticity. For normally distributed and homoscedastic data, one-way ANOVA was used to examine if percent changes in each outcome between baseline to post-intervention were significantly different among groups, and the planned contrasts were performed to examine the difference between intervention and control groups. When the data on the outcomes were not normally distributed, a nonparametric test (the Kruskal-Wallis test) was implemented, and the Mann–Whitney U test was used to analyze the difference between intervention and control groups. The significance level was set to *p* < 0.05 for all the analyses.

## Results

All participants in the HD-tDCS and FCE groups completed the training sessions, and their data were collected successfully (Supplementary Table 1). Twelve participants received 2 mA of HD-tDCS, and no side effects or risk events were reported. No significant differences in demographics (i.e., year, height, and weight) were observed (*p* = 0.135, 0.272, and 0.256; Table 1).Moreover, the passive kinesthesia thresholds of eversion in the FCE group, the ML average CoG sway velocity in OL_EO in the HD-tDCS group, and the AP average CoG sway velocity in OL_EC in the FCE group were not normally distributed and were thus analyzed by a nonparametric test.

**FIGURE 3:**
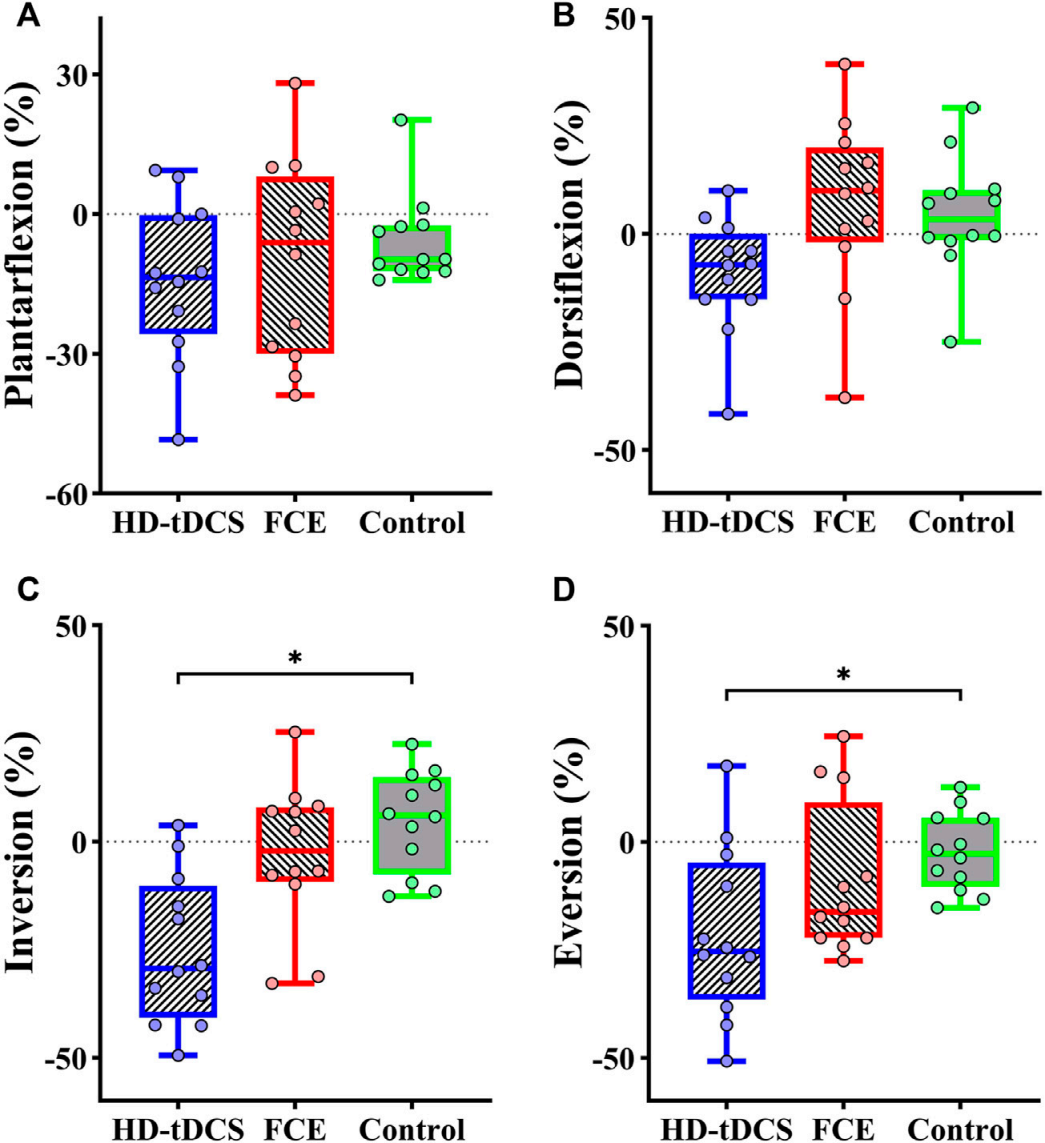
Differences in percent changes in the passive kinesthesia thresholds of **(A)** plantarflexion, **(B)** dorsiflexion, **(C)** inversion, and **(D)** eversion from baseline to post-intervention among groups. HD-tDCS: high-definition transcranial direct current stimulation; FCE: foot core exercise; ^∗^ *p* < 0.05.

Significant differences in the percent changes in the passive kinesthesia thresholds of inversion (*F*_*(2, 32)*_ = 9.210, *p* = 0.001, and $\eta_{p}^{2}$ = 0.365, Table 3) and eversion (H = 7.788, *p* = 0.020) were observed from baseline to post-intervention among groups. Planned contrasts indicated that the HD-tDCS group showed a considerable decrease in the percent change in the passive kinesthesia threshold of inversion (t = − 4.761 and *p* < 0.001, Figure 3C) than the control group. Moreover, the Mann–Whitney U test showed that a greater decrease in percent change in the passive kinesthesia threshold of eversion was observed in the HD-tDCS group than in the control group (*p* = 0.013, Figure 3D). No significant differences were observed for the percent changes in the passive kinesthesia thresholds of plantar flexion (*F*_*(2, 32)*_ = 0.331, *p* = 0.721, and $\eta_{p}^{2}$ = 0.020, Figure 3A) and dorsiflexion (*F*_*(2, 32)*_ = 3.112, *p* = 0.058, and $\eta_{p}^{2}$ = 0.163, Figure 3B).

**TABLE 3:** Percentage changes in passive ankle kinesthesia, foot muscle strength, and one-leg standing balance from baseline to post-intervention.

Outcome (%)	HD-tDCS	FCE	Control	*p*-value
Plantarflexion	− 14.03 ± 16.96	− 9.75 ± 21.24	− 6.48 ± 7.28	0.721
Dorsiflexion	− 9.32 ± 13.50	7.17 ± 20.02	4.31 ± 13.62	0.058
Inversion	− 25.15 ± 17.22	− 2.97 ± 16.82	4.85 ± 11.64	**0.001**
Eversion	− 21.46 ± 19.61	− 9.18 ± 17.70	− 2.32 ± 9.06	**0.020**
MPJ flexor strength	13.77 ± 13.97	11.34 ± 14.30	− 0.13 ± 6.25	**0.029**
Toe flexor strength	9.09 ± 13.73	13.13 ± 14.23	0.74 ± 5.28	**0.047**
ML CoG sway velocity in OL_EO	3.18 ± 14.75	− 10.79 ± 16.51	− 1.35 ± 11.82	**0.020**
AP CoG sway velocity in OL_EO	− 0.93 ± 10.97	− 10.96 ± 12.13	− 7.31 ± 17.05	0.323
ML CoG sway velocity in OL_EC	− 4.14 ± 18.13	− 13.71 ± 18.74	0.28 ± 9.49	0.078
AP CoG sway velocity in OL_EC	− 4.92 ± 20.94	− 17.89 ± 12.82	2.18 ± 7.19	**0.003**

Notes: OL_EO, one-leg standing with eyes open; OL_EC, one-leg standing with eyes closed; ML, medial–lateral; AP, anteroposterior; CoG, the center of gravity; HD-tDCS, high-definition transcranial direct current stimulation; MPJ, metatarsophalangeal joint; FCE, foot core exercise. The bold p-value means *p* < 0.05.

**FIGURE 4:**
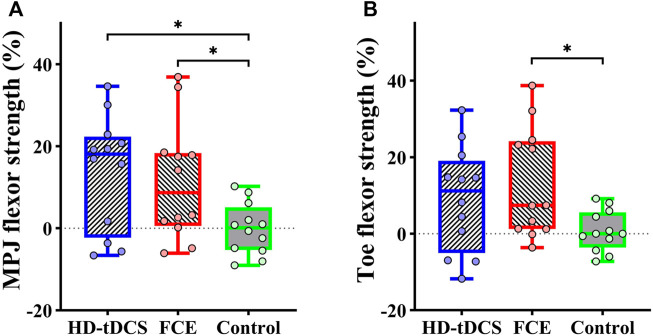
Differences in percent changes in **(A)** the MPJ flexor strength and **(B)** the toe flexor strength from baseline to post-intervention among groups. HD-tDCS: high-definition transcranial direct current stimulation; MPJ: metatarsophalangeal joint; FCE: foot core exercise; ^∗^ *p* < 0.05.

Significant differences in the percent changes in the MPJ flexor strength (*F*_*(2, 32)*_ = 3.979, *p* = 0.029, and $\eta_{p}^{2}$ = 0.199) and toe flexor strength (*F*_*(2, 32)*_ = 3.372, *p* = 0.047, and $\eta_{p}^{2}$ = 0.174) were observed (Table 3). Planned contrasts showed that compared with the control group, a significantly higher increase in percentage change in the MPJ flexor strength was demonstrated in the HD-tDCS group (t = 2.814 and *p* = 0.008, Figure 4A) and in the FCE group (t = 2.323 and *p* = 0.027, Figures 4A), significantly greater increase in the percentage change in toe flexor strength was observed in the FCE group (t = 2.569, *p* = 0.015, Figure 4B).

**FIGURE 5:**
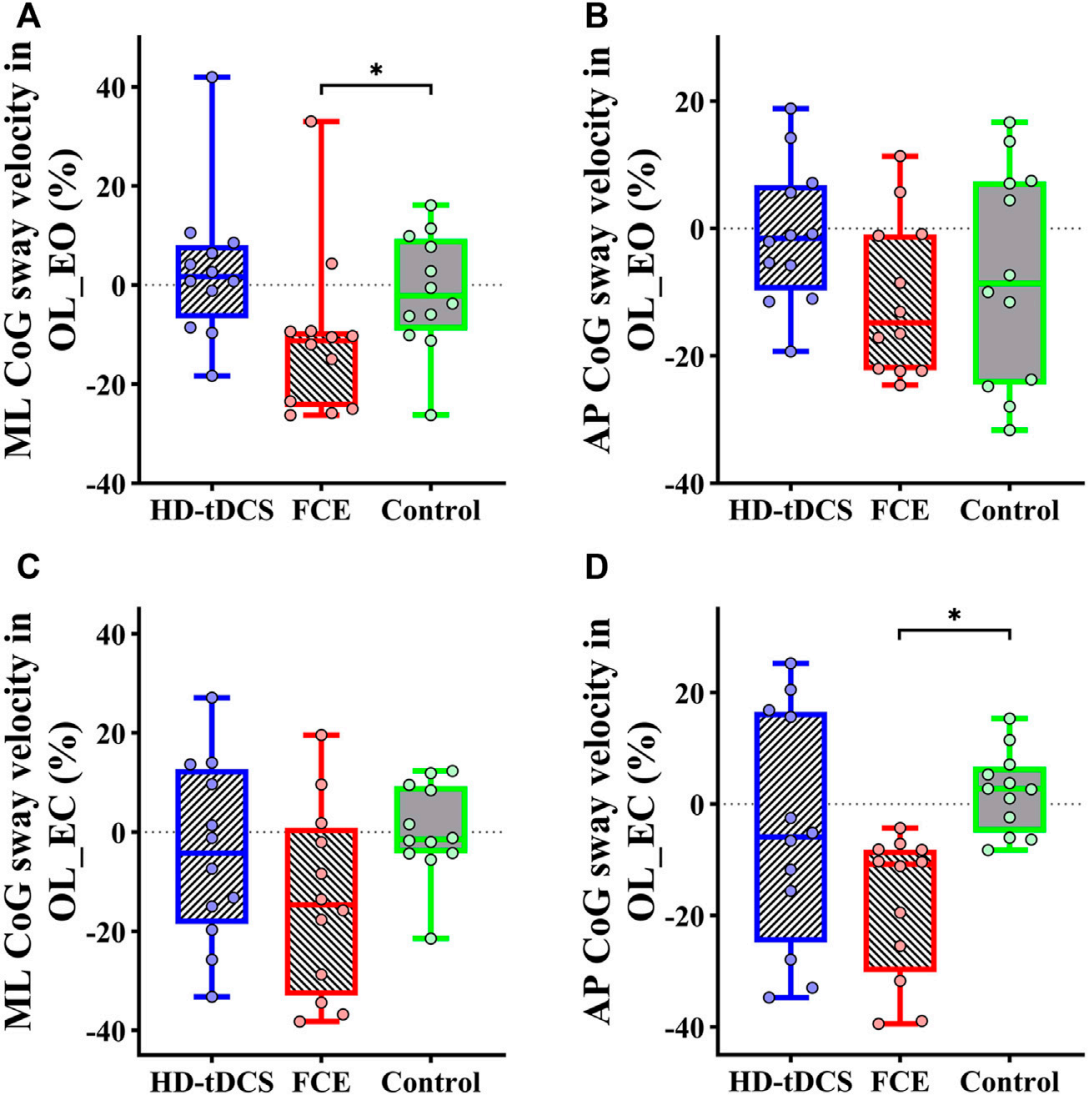
Differences in percent changes in one-leg standing balance from baseline to post-intervention among groups. **(A)** ML CoG sway velocity in OL_EO; **(B)** AP CoG sway velocity in OL_EO; **(C)** ML CoG sway velocity in OL_EC; **(D)** AP CoG sway velocity in OL_EO. OL_EO: one-leg standing with eyes open; OL_EC: one-leg standing with eyes closed; ML: medial–lateral; AP: anteroposterior; CoG, center of gravity; HD-tDCS: high-definition transcranial direct current stimulation; FCE: foot core exercise; ^∗^ *p* < 0.05.

Regarding postural control, significant differences in the percent changes in the ML average CoG sway velocity in OL_EO (H = 7.826 and *p* = 0.020) and the AP average CoG sway velocity in OL_EC (H = 11.740 and *p* = 0.003) were observed (Table 3). Furthermore, the Mann–Whitney U test showed that compared with the control group, FCE induced a greater decrease in the percent changes in the ML average CoG sway velocity in OL_EO (*p* = 0.033, Figure 5A) and the AP average CoG sway velocity in OL_EC (*p* < 0.001, Figure 5D). Moreover, no significant differences were observed for the percent changes in the AP CoG sway velocity in OL_EO (*F*_*(2, 32)*_ = 1.172, *p* = 0.323, and $\eta_{p}^{2}$ = 0.068, Figure 5B) and the ML CoG sway velocity in OL_EC (*F*_*(2, 32)*_ = 2.762, *p* = 0.078, and $\eta_{p}^{2}$ = 0.147, Figure 5C).

## Discussion

This study explored the effects of FCE and HD-tDCS on foot sensorimotor function (i.e., toe flexor strength and passive ankle kinesthesia) and postural control performance. It is observed here that compared with control, HD-tDCS can significantly decrease the passive kinesthesia thresholds of inversion and eversion and improve MPJ flexor strength; on the other hand, compared with control, FCE can enhance the foot muscle strength and decrease the ML average CoG sway velocity in OL_EO and the AP average CoG sway velocity in OL_EC. These results suggested that compared with FCE, HD-tDCS can at least induce comparable, but to some extent distinct, benefits for foot sensorimotor function, suggesting the intervention of cortical modulation *via* HD-tDCS, may uniquely contribute to the enhancement of foot function and postural control, in addition to the intervention targeting central and peripheral function. Future studies are needed to explicitly examine the potential mechanisms underlying the difference between such benefits from tDCS and FCE.

As a novel “brain training”, the current literature provided interesting insights into the potential for tDCS to enhance physical performance, including the sensory function, muscle strength, and balance (Vaseghi et al., 2014; Lattari et al., 2018; de Moura et al., 2019). In this study, the results showed that compared with the control group, the HD-tDCS group demonstrated significantly decreased passive kinesthesia thresholds of ankle inversion and eversion, suggesting that HD-tDCS could improve the sensory perception of the ankle. The previous study showed that tDCS could decrease the vibrotactile threshold of the foot sole when standing and the tactile threshold of the left-center of the distal pulp of the hallux, indicating that tDCS could enhance the ankle-foot somatosensory function (Zhou et al., 2018; Yamamoto et al., 2020). Evidence from neuroimaging studies showed that tDCS increased activation of the left posterior paracentral lobule (including S1) in response to relatively large and easily-perceivable pressure stimuli applied to the right foot sole (Wang et al., 2015). Taken together, these findings suggested that HD-tDCS applied over the sensorimotor cortex can lower the ankle passive kinesthesia thresholds by facilitating the activation of the brain’s somatosensory cortical network.

No significant differences were observed in the plantarflexion and dorsiflexion kinesthesia thresholds in the HD-tDCS group. The neutral regulation effect of tDCS may be related to sensory sensitivity, and healthy participants can accurately perceive the trivial changes in ankle plantarflexion and dorsiflexion (Sun et al., 2015). Thus, it is speculated that the available effect of tDCS-induced improvements in the plantarflexion and dorsiflexion kinesthesia may have been limited. In a word, HD-tDCS helped to decrease passive kinesthesia thresholds of inversion and eversion, implying that the ability to cope with lateral slanted terrain could be improved by multiple sessions of HD-tDCS. On the other hand, no such effects were observed in the FCE group. A potential reason is that FCE targets the central and peripheral components in the somatosensory circuit simultaneously, and its benefits oftentimes can only arise chronically, so the current design of FCE of 4 weeks may not be sufficient to induce significant improvement in the function of mechanoreceptors on the feet, and thus in the sensorimotor function.

It was also observed that 4 weeks of HD-tDCS could enhance MPJ flexor strength and FCE could improve foot muscle strength including MPJ flexor strength and toe flexor strength, indicating HD-tDCS and FCE both promote intrinsic foot muscle strength. In general, anodal HD-tDCS alters the resting membrane potential of the targeted neurons to increase cortical excitability, and these effects can sustain about 6 h after receiving HD-tDCS (Nitsche and Paulus, 2001; Kuo et al., 2013). Moreover, this lasting effect induced by tDCS may continuously reduce short-interval intracortical inhibition, assisting in the production of voluntary force output by modulating descending excitatory drive, thereby improving muscle strength (Nitsche et al., 2005; Weier et al., 2012). FCE is one type of intervention that involves the activation of both central and peripheral systems, which can directly strengthen muscle functions. Specifically, exercises (e.g., short foot exercise and towel curls) included in FCE can activate various flexor muscle activities. Towel curls tend to recruit the flexor hallucis and digitorum longus, and short foot exercise targets the plantar intrinsic muscles of the foot. Thus, studies have shown that FCE targeting the foot muscle strengthening can improve and activate the function in the foot muscles, such as abductor hallucis, flexor digitorum brevis, and flexor hallucis brevis (Fraser and Hertel, 2019; Taddei et al., 2020).

The results of this study also showed that FCE decreased the average CoG sway velocity in one-leg standing balance, which is consistent with previous studies that observed the improvements induced by 4 weeks of short foot exercise in the intrinsic foot muscle performance during a static unilateral balancing activity and in the performance of functional balance task (Mulligan and Cook, 2013). Moreover, short foot exercise and towel curl could decrease the ML center of pressure movement on the dominant limb by a small amount (≈5 mm) during a dynamic-balance test (Lynn et al., 2012). Taken together, our results suggested that FCE is of great promise to improve the postural control performance of one-leg standing. However, no significant differences were observed for the CoG sway velocity in the OL_EO condition. One possible explanation is that visual information has a main effect on the standing balance with eyes open, and thus, maintaining the balance does not need more information integrated by the sensorimotor cortex.

Moreover, no significant improvements in postural control as induced by HD-tDCS were observed. Although a meta-analysis revealed that tDCS applied over M1 appeared to improve postural control, more recent studies also showed significant effects of tDCS targeting M1 on standing postural control (de Moura et al., 2019; Zhou et al., 2021). One possible reason may be that the leg area of M1 is located in parallel to the direction of current flow delivered by tDCS so that only a very small electric field can be generated over M1. Therefore, the excitability of M1 can be increased to a very limited extent, which largely limits the effects of tDCS targeting M1 on standing postural control performance (Zhou et al., 2021). Future studies are highly demanded to optimize the tDCS montages targeting M1 to maximize the activation of this important brain region.

The mechanisms underlying the benefits of HD-tDCS and FCE are distinct, and the dose-response relationship in these two interventions may thus be different. Therefore, one session of FCE may induce a different response from tDCS of the same time length. Future studies implementing multiple assessments in the postural control performance (e.g., assessing every week through the intervention) may provide important knowledge for the dose-response relationships of the two interventions, and then, we can more explicitly examine and compare the efficacy of these two interventions.

This study is of several limitations in addition to the uncertain difference in the dose-response relationship between tDCS and FCE. Only a small sample of male adults was enrolled, future studies with a larger sample size of participants with similar numbers of men and women are needed. Moreover, healthy young adults were recruited, and the effects of HD-tDCS and FCE on foot sensorimotor function and postural control in populations with diminished or impaired foot functionality are worth to be examined. Also, no follow-up assessment was conducted in this study to investigate the lasting effect of the interventions. Finally, the positive effects of HD-tDCS may be limited by a 4-week intervention with a rather long interval (i.e., three sessions weekly). Notably, the focalization degree of HD-tDCS here was not measured quantitatively and should be explicitly assessed in future studies, which can provide helpful knowledge for the design of sensorimotor tDCS.

## Conclusion

This study demonstrated that 4 weeks of HD-tDCS and FCE induced distinct benefits on foot sensorimotor function and the standing postural control performance in healthy young adults. HD-tDCS could improve foot sensorimotor function including the MPJ flexor strength and passive kinesthesia thresholds of ankle inversion and eversion. Meanwhile, FCE could also strengthen foot muscle strength and enhance postural control performance in one-leg standing. It indicates that for the rehabilitation of the foot sensorimotor function and standing postural control, interventions targeting the peripheral and the central components are both needed. Future studies are warranted to further explore the potential benefits of implementing the combined type of intervention (i.e., interventions combining both FCE and tDCS).

## Data Availability

The original contributions presented in the study are included in the article/Supplementary Material, further inquiries can be directed to the corresponding authors.
