# Supplementary material for: Effects of 4 Weeks of High-Definition Transcranial Direct Stimulation and Foot Core Exercise on Foot Sensorimotor Function and Postural Control
Source: Front Bioeng Biotechnol. 2022 Jun 1;10:894131. doi: 10.3389/fbioe.2022.894131 (PMC9198259; doi:10.3389/fbioe.2022.894131)
Supplement: Supplementary file 1 [file Table1.DOCX]

Supplementary Material

# Supplementary Table 1. Effects of HD-tDCS and FCE on passive ankle kinesthesia, foot muscle strength, and postural control

| Outcomes | HD-tDCS group | | FCE group | | Control group | |
| --- | --- | --- | --- | --- | --- | --- |
|  | pre | post | pre | post | pre | post |
| Plantarflexion (°) | 1.44 ± 0.80 | 1.14 ± 0.35 | 1.43 ± 0.58 | 1.21 ± 0.34 | 1.14 ± 0.30 | 1.07 ± 0.29 |
| Dorsiflexion (°) | 1.43 ± 0.45 | 1.27 ± 0.38 | 1.33 ± 0.40 | 1.40 ± 0.44 | 1.24 ± 0.33 | 1.27 ± 0.27 |
| Inversion (°) | 2.95 ± 1.07 | 2.11 ± 0.56 | 2.52 ± 1.05 | 2.36 ± 0.87 | 2.17 ± 0.73 | 2.24 ± 0.66 |
| Eversion (°) | 3.18 ± 1.17 | 2.41 ± 0.81 | 3.14 ± 0.99 | 2.78 ± 0.76 | 2.26 ± 0.50 | 2.19 ± 0.44 |
| MPJ flexor strength (N/kg) | 3.63 ± 1.21 | 4.17 ± 1.71 | 3.03 ± 0.87 | 3.37 ± 1.05 | 2.91 ± 0.99 | 2.88 ± 0.95 |
| Toe flexor strength (N/kg) | 1.76 ± 0.49 | 1.90 ± 0.52 | 1.76 ± 0.70 | 1.99 ± 0.79 | 1.72 ± 0.72 | 1.74 ± 0.74 |
| ML CoG sway velocity in OL_EC (mm/s) | 18.78 ± 4.13 | 19.49 ± 5.64 | 22.54 ± 5.16 | 19.80 ± 4.24 | 27.93 ± 9.32 | 27.68 ± 10.68 |
| AP CoG sway velocity in OL_EC (mm/s) | 29.98 ± 6.73 | 29.88 ± 8.46 | 36.18 ± 9.09 | 32.10 ± 8.91 | 31.26 ± 5.88 | 29.24 ± 8.65 |
| ML CoG sway velocity in OL_EO (mm/s) | 35.33 ± 10.03 | 33.81 ± 11.74 | 41.88 ± 11.47 | 34.89 ± 7.29 | 48.06 ± 18.59 | 48.22 ± 19.15 |
| AP CoG sway velocity in OL_EO (mm/s) | 57.50 ± 10.50 | 54.79 ± 16.26 | 70.65 ± 15.83 | 56.94 ± 9.82 | 58.80 ± 15.85 | 59.48 ± 14.25 |

Notes: OL_EO: one-leg standing with eyes open; OL_EC: one-leg standing with eyes closed; ML: medial-lateral; AP: anteroposterior; CoG, the center of gravity; HD-tDCS: high-definition transcranial direct current stimulation; MPJ: metatarsophalangeal joint; FCE: foot core exercise.
